# Minutes to Save a Life in Acute GI Bleeding: A Systematic Review of Early vs. Delayed Endoscopy

**DOI:** 10.7759/cureus.100176

**Published:** 2025-12-27

**Authors:** Sara Abdalaziz Mohammed Ibrahim, Hamza Abubaker Abdin Sidahmed, Israa Elfadil Ali Ahmed, Qays-Marcel Ghneim, Elkhansaa A Elsamani, Asim Ahmed

**Affiliations:** 1 Internal Medicine, Healthline Medical Group, Abu Dhabi, ARE; 2 Medical Claims, Almadallah Healthcare Management, Dubai, ARE; 3 Primary and Urgent Care, Albadaya General Hospital, Al Badayea, SAU; 4 General Medicine, Soroka Medical Center, Be’er Sheva, ISR; 5 Internal Medicine, University Hospital Galway, Galway, IRL; 6 Medicine and Surgery, University of Gezira, Wad Madani, SDN

**Keywords:** early endoscopy, endoscopy timing, non-variceal upper gastrointestinal bleeding, systematic review, urgent endoscopy

## Abstract

Non-variceal upper gastrointestinal bleeding (NVUGIB) is a common and potentially life-threatening emergency that presents major clinical and logistical challenges worldwide, making the timing of endoscopic management particularly important. Endoscopy remains the cornerstone of diagnosis and therapy in NVUGIB, allowing identification of the bleeding source and delivery of hemostatic therapy, yet the optimal timing, urgent within 6-12 hours versus early within 24 hours, continues to be debated, particularly when deciding between hemodynamically unstable or otherwise high-risk patients and more stable, low-risk presentations. This systematic review, conducted in accordance with Preferred Reporting Items for Systematic reviews and Meta-Analyses (PRISMA) 2020 guidelines, evaluated the effect of endoscopy timing on mortality, rebleeding, therapeutic interventions, and health-system outcomes, with special consideration for elderly and anticoagulated patients, in whom endoscopy may be more challenging. Evidence synthesized from randomized trials, cohort studies, and large database analyses indicates that early endoscopy within 24 hours is consistently associated with shorter hospital stays, lower costs, and better discharge outcomes, particularly in patients at higher clinical risk. These differences likely reflect the divergent risk profiles of high-risk compared with low-risk patients. While urgent endoscopy within 6-12 hours may offer advantages in high-risk or hemodynamically unstable patients, routine use in low-risk cases does not improve survival. It may also increase rebleeding. Overall, performing endoscopy within 24 hours provides the best balance of safety and efficiency and should be considered the preferred standard of care, with urgent procedures reserved for selected high-risk patients.

## Introduction and background

Non-variceal upper gastrointestinal bleeding (NVUGIB) is one of the most frequent and challenging emergencies in gastroenterology [1]. It accounts for nearly 300,000 hospital admissions annually in the United States and carries a mortality rate ranging from 3% to 14%, despite advances in diagnostic and therapeutic modalities [2], underscoring its substantial impact on healthcare systems. NVUGIB imposes a substantial healthcare burden due to high recurrence rates, frequent transfusion requirements, and prolonged hospitalizations [3].

Timely endoscopic evaluation and intervention remain central to NVUGIB management, serving both diagnostic and therapeutic purposes [4]. Esophagogastroduodenoscopy (EGD) enables direct visualization of bleeding lesions and facilitates risk stratification based on stigmata of recent hemorrhage. It also allows immediate therapeutic maneuvers such as injection therapy, thermal coagulation, or mechanical hemostasis. Current guidelines generally recommend that endoscopy be performed within 24 hours of presentation in most patients with suspected NVUGIB [5]. However, considerable debate persists as to whether urgent intervention (within 6-12 hours) confers additional benefits compared to early but non-immediate procedures performed within 24 hours, particularly when balancing potential advantages in high-risk patients against limited added value and resource use in low-risk cases [6].

Evidence has demonstrated reductions in hospital stay, resource utilization, and healthcare costs when endoscopy is performed within 24 hours [7]. Nevertheless, findings regarding mortality reduction remain inconsistent, with some studies showing no clear additional survival advantage of very urgent procedures [8]. In many clinical scenarios, patient-level factors including hemodynamic instability, advanced age, comorbidities, and anticoagulant use are often stronger determinants of prognosis than the precise timing of endoscopy [9]. Moreover, adherence to guideline-recommended practices varies across regions and institutions, influencing outcomes and contributing to disparities in care [10].

Peptic ulcer disease remains a major cause of NVUGIB worldwide and continues to pose significant clinical and economic challenges despite advances in medical and endoscopic therapies, including declining *Helicobacter pylori *prevalence and increased proton pump inhibitor (PPI) use in many regions [11]. The introduction of PPIs and refinements in endoscopic hemostasis techniques have improved management; however, rebleeding remains a major concern, prolonging hospitalization and increasing morbidity, particularly in high-risk presentations. Mortality rates are particularly high among patients presenting with severe hemodynamic instability or profound anemia, with case fatality approaching 40% in such settings [12].

Elderly patients represent a particularly vulnerable subgroup. Frailty, multiple comorbidities, and polypharmacy, including anticoagulant therapy, often worsen prognosis and complicate therapeutic decision-making. This underscores the need for individualized management strategies that carefully balance the risks and benefits of endoscopy timing and are guided by dynamic clinical parameters rather than fixed risk categories. While effective care generally combines pharmacological stabilization with endoscopic hemostasis, debate persists as to whether urgent intervention offers meaningful advantages over early procedures [13].

Available evidence suggests that very urgent endoscopy (≤6-12 hours) does not consistently translate into superior outcomes. In some cases, early clot manipulation may paradoxically increase the risk of rebleeding, particularly in hemodynamically stable or low-risk patients, likely reflecting interactions between clot stability and underlying hemodynamic status [14]. Conversely, delaying endoscopy beyond 24 hours has been consistently associated with adverse outcomes, including higher mortality rates and increased healthcare costs. These findings emphasize the importance of tailoring timing strategies to patient risk profiles rather than applying a uniform approach. Overall, the literature highlights both the potential benefits and the limitations of urgent endoscopy, reinforcing the need for systematic synthesis to clarify its impact across diverse patient populations [15].

Against this background, the present systematic review provides a comprehensive synthesis of the available evidence to clarify how different endoscopy timing thresholds, such as urgent (≤6-12 hours), early (≤24 hours), and delayed (>24 hours), influence mortality, rebleeding, therapeutic interventions, and health-system outcomes in adults with NVUGIB, and to inform clinical decision-making and future guideline development.

Objectives

This systematic review aimed to comprehensively evaluate the impact of endoscopy timing in patients with non-variceal upper gastrointestinal bleeding. Specifically, it sought to determine whether urgent procedures (≤6-12 hours) offer advantages over early (≤24 hours) and delayed (>24 hours) interventions in reducing mortality, recognizing that these timing categories, as used in the primary studies, may be subject to selection and confounding biases. In addition, the review examined the effect of timing on rebleeding rates and the need for therapeutic interventions, with attention to differences between high-risk and low-risk subgroups. It further assessed the influence of early endoscopy on health-system outcomes, including hospital length of stay, intensive care utilization, costs, and discharge disposition, as key indicators of resource use and overall quality of hospital care. Special populations, such as elderly patients and those receiving anticoagulant therapy, were also considered to clarify risks, benefits, and tailored management strategies. Finally, the review integrated evidence from randomized controlled trials (RCTs), nationwide databases, and hospital-based cohorts to provide a comprehensive synthesis that informs guideline recommendations for optimal endoscopy timing.

## Review

Methods

This systematic review was conducted in accordance with the Preferred Reporting Items for Systematic Reviews and Meta-Analyses (PRISMA) 2020 guidelines. A protocol was developed in advance to define eligibility criteria, search strategy, screening procedures, data extraction, and synthesis methods. Although the review was not formally registered in the International Prospective Register of Systematic Reviews (PROSPERO), all methodological steps were predefined in a written protocol and consistently applied.

Eligibility Criteria

Studies were eligible if they included adult patients presenting with NVUGIB and evaluated outcomes related to the timing of endoscopy. Timing was categorized as urgent (≤6-12 hours), early (≤24 hours), or delayed (>24 hours). Eligible outcomes included mortality, rebleeding, need for therapeutic intervention, length of hospital stay, healthcare costs, intensive care utilization, and discharge disposition. We included RCTs, prospective and retrospective cohort studies, case control studies, nationwide administrative database analyses, and systematic reviews with meta-analyses. Exclusion criteria were studies restricted to variceal bleeding without stratified NVUGIB data, pediatric populations, case series with fewer than ten patients, and commentaries or abstracts lacking extractable data.

Search Strategy

A comprehensive literature search was conducted in PubMed, Scopus, Web of Science, and the Cochrane Library for the period January 2000 to September 2025. The strategy combined Medical Subject Headings (MeSH) and free-text terms to maximize search sensitivity across databases, including “upper gastrointestinal bleeding”, “UGIB”, “nonvariceal bleeding”, “endoscopy”,“esophagogastroduodensoscopy”, “timing”, “urgent”, “early”, and “delayed”. Equivalent strategies were adapted for each database. Reference lists of included studies and relevant reviews were also screened to identify additional records.

Study Selection

All retrieved records were imported into EndNote X9 (Clarivate Plc, London, United Kingdom), and duplicates were removed. Two reviewers independently screened titles and abstracts, followed by full-text assessment of potentially eligible articles. Disagreements were resolved through discussion or by consulting a senior reviewer.

Data Extraction

Two reviewers independently extracted data using a standardized form. Extracted information included first author, year of publication, study country and setting, design, sample size, patient population, definitions of endoscopy timing, reported outcomes, and quality appraisal. Discrepancies were resolved by consensus after discussion, with a senior reviewer consulted when needed.

Risk of Bias Assessment

RCTs were assessed using the Cochrane Risk of Bias 2.0 tool (Cochrane, London, United Kingdom), while observational cohort and case-control studies were appraised with the Newcastle-Ottawa Scale (NOS). Nationwide database studies and quasi-experimental designs were evaluated with adapted NOS criteria focusing on selection, comparability, and outcome assessment. Systematic reviews and meta-analyses were assessed with the AMSTAR-2 (A MeaSurement Tool to Assess systematic Reviews 2) checklist for methodological quality. This differentiated, design-specific approach to risk-of-bias assessment was intended to enhance the robustness of the overall methodological appraisal. Studies were categorized as having low, moderate, or high risk of bias, with abstract-only reports considered higher risk due to limited methodological detail.

Data Synthesis

Because of heterogeneity in study designs, outcome definitions, and reporting methods, a quantitative meta-analysis was not feasible. Instead, findings were synthesized narratively. Results were organized under five predefined objectives: mortality, rebleeding and therapeutic interventions, health-system outcomes, special populations, and integration of evidence across study designs. Where applicable, pooled estimates from previously published meta-analyses were incorporated to support the overall synthesis.

Result

Study Selection

**Figure 1:**
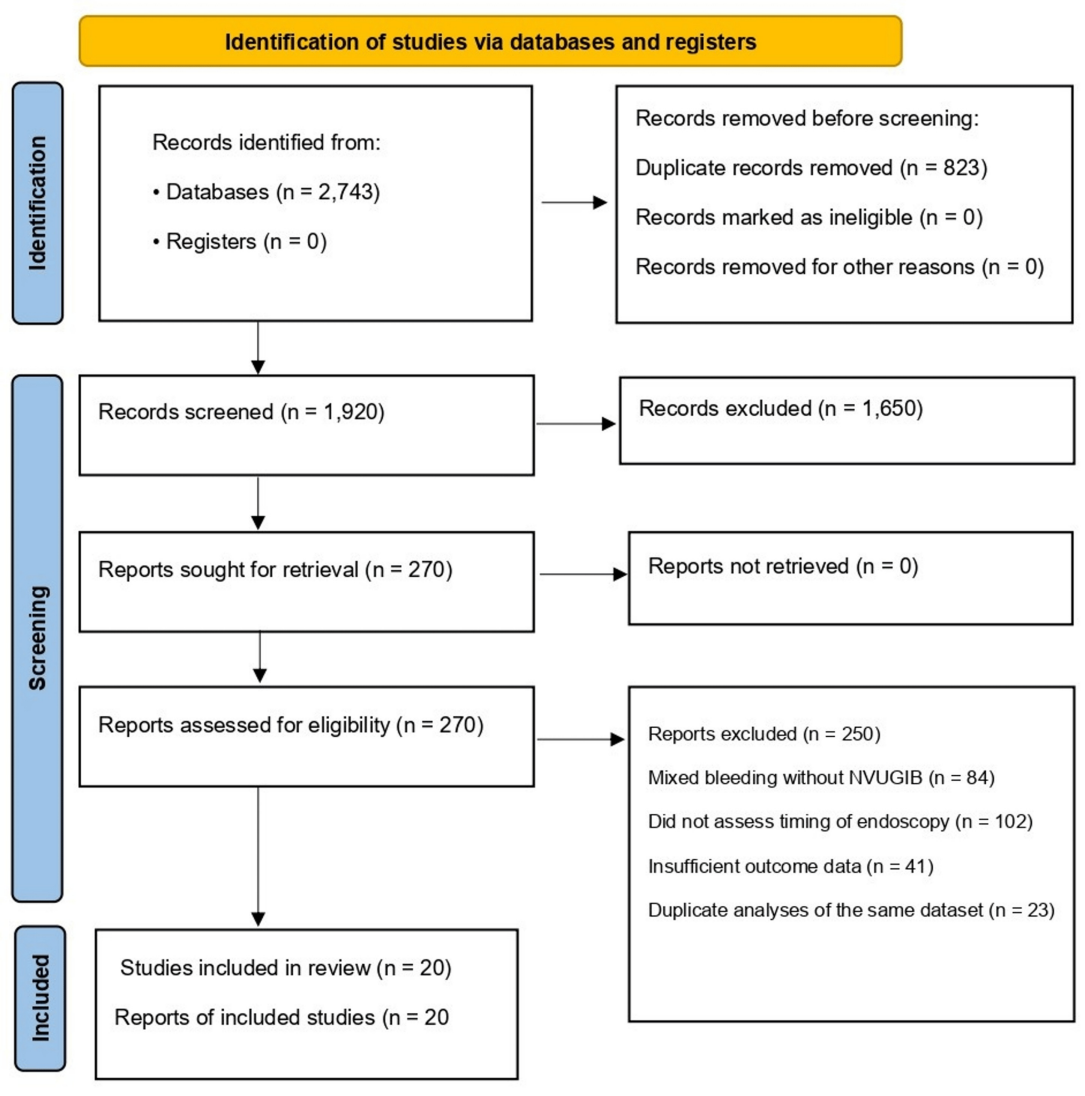
PRISMA flow diagram NVUGIB, non-variceal upper gastrointestinal bleeding; PRISMA, Preferred Reporting Items for Systematic Reviews and Meta-Analyses

A total of 2,743 records were identified from database searching (PubMed = 1,125, Scopus = 912, Web of Science = 496, Cochrane Library = 210). After removing 823 duplicates, 1,920 unique records remained for screening. Title/abstract screening excluded 1,650 records as irrelevant, most commonly because they focused on variceal bleeding, pediatric populations, non-endoscopic interventions, or conference announcements without usable outcome data. Full texts were retrieved for 270 articles. Of these, 250 were excluded because 84 involved variceal or mixed bleeding populations without stratified NVUGIB results, 102 did not analyze endoscopy timing as an exposure, 41 had insufficient outcome data (or were descriptive only), and 23 were duplicate analyses of the same dataset. Overall, the dominant full-text exclusion drivers were a lack of stratified NVUGIB data and the absence of an endoscopy-timing analysis. The selection process is shown in the PRISMA flow diagram (Figure 1).

Overview of Included Studies

Ultimately, 20 studies met the inclusion criteria and were included in the review [16-35], spanning complementary designs and populations across controlled and real-world settings. This evidence base included randomized trial data (e.g., Lau et al., 2020 [20]), large nationwide database analyses (e.g., Weissman et al., 2023 [18]), propensity-score-matched/quasi-experimental cohorts, and single- or multicenter hospital cohorts. Prospective audit/registry-style evidence (e.g., Siau et al., 2019 [32]) further supported the interpretation of timing patterns in routine practice. Overall, the methodological breadth strengthens external validity while retaining clinically granular cohort-level insights (Table 1).

**Table 1:** Characteristics and quality appraisal of included studies evaluating endoscopy timing in NVUGIB. Studies are summarized by country/setting, design, sample size, population, timing thresholds used for comparison, key outcome findings, and an overall quality/risk-of-bias judgement as assessed by the review team. Timing categories (e.g., ≤6 h, ≤12 h, ≤24 h, >24 h) reflect each study’s own operational definitions and are reported as presented in the original articles. UGIB, upper gastrointestinal bleeding; NVUGIB, non-variceal upper gastrointestinal bleeding; EGD, esophagogastroduodenoscopy; ED, emergency department; ICU, intensive care unit; LOS, length of stay; RCT, randomized controlled trial; PSM, propensity score matching; IPTW, inverse probability of treatment weighting; GBS, Glasgow–Blatchford score; OR, odds ratio; aOR, adjusted odds ratiol NSAID, non-steroidal anti-inflammatory drug; UGIE, upper gastrointestinal endoscopy

ID	Study & Reference	Country (Setting)	Design	Sample Size (N)	Population	Intervention / Comparison	Key Findings	Quality Assessment
1	Guo et al., 2022 [16]	Hong Kong (Territory-wide hospital system)	Retrospective cohort	6,474	Adults admitted with acute UGIB	Urgent (≤6 hours) vs. Early (6–24 hours) vs. Late (>24 hours) vs. Early (6–24 hours) vs. Late (>24 hours)	Urgent group (6.2%) required repeat procedures more often than the early group (4.3%). Outcomes in late group were poorer than early. No significant mortality difference.	Moderate: Large cohort with strong external validity; limited by retrospective selection bias.
2	Jeong et al., 2019 [17]	South Korea (Single-center ED)	Retrospective cohort	1,101	Adults with acute GI hemorrhage	Early (<24 hours) vs. Delayed (≥24 hours)	Mortality was significantly higher with delay: 6.4% (13/203) vs. 2.8% (25/898). Delay ≥ 24 hours was an independent predictor of death.	Moderate: Adequate sample and adjustment; limited by single-center design.
3	Weissman et al., 2023 [18]	USA (Nationwide Inpatient Sample)	Retrospective nationwide analysis	1,082,516 (553,186 underwent EGD)	Adults with Non-Variceal UGIB (NVUGIB)	EGD timing: ≤24 hours vs. 24–48 hours vs. 48–72 hours vs. >72 hours	Early EGD less than24h) reduced mortality, ICU stays, and costs (P < 0.001). Male sex (OR 1.30), Hispanic race (OR 1.10), and Asian race (aOR 1.38) independently predicted worse outcomes.	Moderate: Massive sample size; limited by potential coding bias and lack of clinical nuances in database.
4	Capela et al., 2024 [19]	Portugal (Single-center cohort)	Retrospective cohort	636 (138 on anticoagulants)	Adults with NVUGIB using anticoagulants	Early (≤24 hours) vs. Delayed (>24 hours)	Early group had higher endoscopic therapy rates (OR 2.4) and shorter stays (7 vs. 9 days). Higher ICU admission in early group (OR 2.7). No 30-day mortality difference.	Moderate: Adjusted analysis; limited by single-center design and small anticoagulant subgroup.
5	Lau et al., 2020 [20]	Hong Kong (China, SAR)	RCT	516, GBS ≥12, urgent ≤6h vs early 6–24h, no mortality benefit.	acute UGIB with Glasgow–Blatchford score ≥12	Urgent endoscopy within 6 hours vs early endoscopy 6–24 hours after gastroenterology consultation	urgent endoscopy did not reduce 30-day mortality vs early (and did not clearly improve rebleeding either)	Moderate: Nationally representative; lack of stratification between variceal and non-variceal types.
6	Tripathi et al., 2020 [21]	USA (National Inpatient Sample)	Quasi-exp, Propensity Score Matched	16,806 (8,403 per group)	Adults with non-variceal UGIB	Early (Day 0–1) vs. Delayed (Day 2)	Early EGD led to shorter LOS (4.02 vs 4.72 days) and lower charges. However, mortality was slightly higher in the early group (1.64% vs 1.04%).	Moderate/High Risk: Robust matching but abstract-only; potential for residual confounding.
7	Cooper et al., 2009 [22]	USA (Medicare 5% sample)	Retrospective population analysis	2,592 (Elderly)	Patients 66 years or older with bleeding peptic ulcers	Early (≤1 day) vs. Delayed (>1 day)	71.5% received early EGD. Associated with shorter stay (-1.95 days) and lower surgery risk (OR 0.37). No mortality difference.	Moderate: Large national dataset; limited by reliance on claims data and lack of clinical detail.
8	El-Dallal et al., 2020 [23]	USA (Nationwide Inpatient Sample)	Retrospective with IPTW adjustment	120,835 (Elderly)	Geriatric patients (65 or older) with NVUGIB	Early (≤24 hours) vs. Late (>24 hours)	Early EGD reduced LOS (-1.17 days) and charges (-5,717). Paradoxically associated with increased mortality (OR 1.32) and higher transfer rates.	Moderate/High Risk: Large database; abstract-only; mortality finding may reflect higher acuity in the early group.
9	Chang et al., 2025 [24]	Thailand (Multicenter)	Retrospective cohort with PSM	730 (556 after matching)	Adults with NVUGIB who underwent EGD	Early (≤24 hours) vs. Late (>24 hours)	Early group showed more high-risk stigmata and required more hemostasis. Lower transfusion needs, shorter LOS, and lower costs. No mortality difference.	Low/Moderate: Robust matching strengthens findings; single-country setting limits generalizability.
10	Arslan et al., 2021 [25]	Turkey (Single-center)	Retrospective cohort	104 (80 early, 24 late)	Adults with NVUGIB	Early (≤24 hours) vs. Late (>24 hours)	Early EGD associated with shorter stay and lower costs. In high-risk patients (GBS 9 or higher), late EGD significantly worsened outcomes.	Moderate: Small sample size and single-center design; retrospective.
11	Garg et al., 2017 [26]	United States	Retrospective longitudinal, administrative database study (2007–2013)	Total UGIB admissions identified: 2,066,707 adults. EGD performed: 1,735,116; No EGD: 331,591. Early EGD (≤24h): 1,020,744; Delayed EGD (>24h): 714,372.	Adults (≥18 years) hospitalized with a primary discharge diagnosis of upper gastrointestinal bleeding (ICD-9-CM coding).	Early EGD (≤24 hours) vs Delayed EGD (>24 hours) vs No EGD	Early EGD was associated with lower in-hospital mortality and morbidity, shorter length of stay, and lower total hospital costs compared with delayed/no EGD (association, not proven causation).	Moderate quality (observational): very large nationwide sample and adjusted analyses, but retrospective design with administrative coding, selection bias/confounding by indication, and limited clinical granularity (e.g., physiology/endoscopic stigmata).
12	Cagir et al., 2024 [27]	Turkey (Tertiary referral hospital)	Retrospective cohort study	468 (260 patients 65 years or older; 208 younger)	Adults with NVUGIB stratified by age (65 or older vs. younger than 65)	Very Early (<12 hours) vs. Early (12–24 hours) vs. Late (>24 hours)	Endoscopy timing did not significantly affect mortality or rebleeding in either age group. However, early EGD was associated with significantly shorter hospital stays and reduced transfusion requirements in the elderly.	Moderate Risk: Single-center retrospective design; age-stratification adds value, but subject to selection bias and confounding.
13	Saleem et al., 2020 [28]	USA (Community hospital)	Retrospective cohort	178	Adults with UGIB (mostly NVUGIB)	Early (<24 hours) vs. Late (>24 hours)	Early EGD significantly reduced LOS (3.8 vs 6.1 days) and 30-day readmission (6.3% vs 16.7%). No diff in mortality.	Moderate: Outcomes clearly reported; limited by small, single-center sample.
14	Güven et al., 2023 [29]	Turkey (Ankara City Hospital)	Retrospective cohort	240	High-risk NVUGIB (GBS) ≥ 12	Urgent (<12 hours) vs. Early (12–24 hours)	No significant difference between urgent (<12 hour) and early (12–24 hour) endoscopy in composite outcomes (mortality/rebleeding/re-intervention/surgery). Early group required more blood transfusion and had longer hospital length of stay.]	Moderate: Targeted high-risk subgroup is a strength; retrospective design.
15	Jairath et al., 2012 [30]	UK (Nationwide Audit)	Prospective nationwide cohort	4,478	Adults with acute NVUGIB	24 hours or less vs. more than 24 hours	Early EGD associated with lower 30-day mortality and reduced surgery risk. Timing <12 hour showed no additional benefit after risk adjustment.	High: Large, prospective, nationwide study; robust methodology.
16	Iqbal et al., 2018 [31]	USA (Single-center)	Retrospective review	372	Adults with acute NVUGIB	Early (<24 hours) vs. Delayed (>24 hours)	Early EGD associated with shorter LOS and reduced costs. Mortality was driven by comorbidities/severity rather than timing.	Moderate: Typical retrospective limitations; potential for selection bias.
17	Siau et al., 2019 [32]	UK (143 hospitals)	Prospective multicentre audit	4,478	Adults with acute UGIB	EGD ≤12 hours, 12–24 hours, >24 hours	Median EGD time was 20 hour. EGD ≤24h reduced LOS but didn't reduce mortality. EGD <12 hour showed no survival benefit.	High quality – large prospective multicentre audit with standardized data collection and broad external validity; however, as an observational audit it remains vulnerable to confounding by indication (sicker patients scoped earlier/later), site-level practice variation, and limited causal inference.
18	Villanueva et al., 2013 [33]	Spain (Multicentre RCT)	RCT	921	Adults with acute UGIB	Restrictive vs Liberal Transfusion	Restrictive strategy (Hb <7) reduced 45-day mortality (5% vs 9%) and rebleeding.	Very high quality – well-conducted multicentre randomized controlled trial with strong internal validity and clinically meaningful endpoints; limitation is indirectness for your review question because it evaluates transfusion strategy (timing of endoscopy was protocolized and not the randomized exposure).
19	Barbu et al., 2025 [34]	Romania	Retrospective cohort	N = 364.	Adults (≥18 years) hospitalized with confirmed NVUGIB based on clinical presentation, labs, and UGIE; excluded variceal UGIB, lower GI bleeding, incomplete records, or major confounding comorbidities.	All patients underwent UGIE within 24 hours; endoscopic therapy included adrenaline injection and hemoclips; surgery for failed/recurrent bleeding. Descriptive comparison to historical national/international literature; no survival analysis due to data structure.	Predominant etiologies: peptic ulcers, erosive gastritis, Mallory–Weiss syndrome, gastric neoplasms. Major risk factors: NSAID use, oral anticoagulation, alcohol. Endoscopic hemostasis achieved in most cases; surgery required in 11.5% (refractory/recurrent bleeding). Overall mortality 10.9%..	Moderate quality / moderate risk of bias: consecutive real-world cohort with explicit criteria, but retrospective single-center design; medication exposure details (dose/duration/concomitant use) not consistently captured; no time-to-event/survival analysis.
20	Lucas Ramos et al., 2023 [35]	Spain (La Paz University Hospital)	Prospective observational	1,096 (682 Urgent vs. 414 Early)	Adults with suspected acute upper GI bleeding	Urgent (<6 hour) vs. Early (6–24 hour)	No significant difference in mortality (5% vs. 7.7%) or rebleeding. However, Urgent EGD (<6 hour) significantly reduced transfusion needs and predicted lower mortality specifically in those with high-risk lesions (Forrest I–IIB).	Moderate – large prospective observational cohort, but single-center and non-randomized; timing likely influenced by clinical severity (confounding by indication), with potential selection bias and residual confounding despite subgroup analyses.

Synthesis of Findings

Across the 20 included studies, the evidence base encompassed millions of NVUGIB admissions, with the clearest and most consistent signals derived from large nationwide datasets evaluating “early” endoscopy (≤24 hours). In Garg et al.'s study (2,066,707 admissions) [26] and Weissman et al.'s study (1,082,516 admissions) [18], endoscopy within 24 hours was consistently associated with lower inpatient mortality and more efficient care (shorter length of stay and lower resource use). By contrast, several cohort-level and territory-wide datasets suggest that advancing from “early” (6-24 hours) to “very urgent” (≤6-12 hours) does not reliably add survival benefit once resuscitation and stabilization are accounted for. Guo et al. reported superior outcomes with endoscopy within 24 hours compared with later timing, while the most urgent timing did not clearly outperform the 6-24-hour window after risk adjustment [16]. Jeong et al. similarly reinforced harm signals with delays beyond 24 hours [17].

Smaller and moderate-sized cohorts consistently supported efficiency gains (e.g., shorter length of stay and lower costs), while mortality effects were more variable and often limited by residual confounding and lower event counts [24,25]. This pattern was also seen in clinically targeted cohorts such as anticoagulant-exposed populations [19] and general hospital cohorts [28,31].

Subgroup analyses added practical nuance. In high-risk NVUGIB cohorts (e.g., elevated Glasgow-Blatchford scores), Güven et al. suggested early endoscopy is feasible and safe [29]. Anticoagulation-focused evidence indicated early endoscopy can be delivered without excess harm in anticoagulant-exposed patients [19], and anticoagulation status did not negate early-EGD benefit signals in large administrative datasets [18].

**Figure 2:**
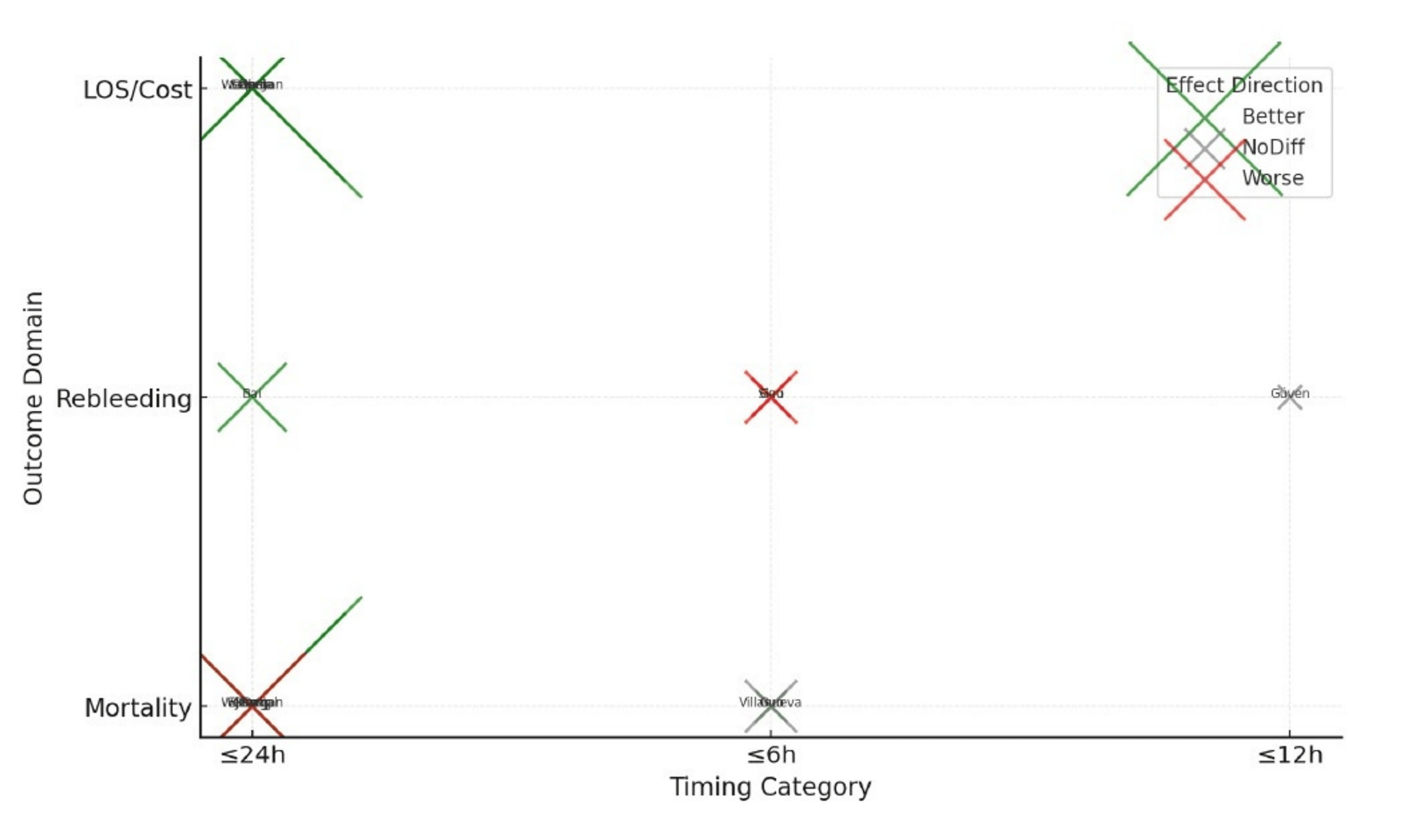
Summary of outcome patterns across objectives in the included studies of NVUGIB, stratified by outcome domain and timing threshold. Direction of effect of endoscopy timing across outcome domains. Key large datasets: [26,18] LOS, length of stay; EGD, esophagogastroduodenoscopy; NVUGIB, non-variceal upper gastrointestinal bleeding

Beyond timing alone, co-interventions influence outcomes and interpretation. Villanueva et al. demonstrated improved outcomes under a restrictive transfusion strategy, highlighting the importance of pathway-level management alongside timing [33]. Pooled evidence syntheses concluded that ≤24-hour endoscopy most consistently improves efficiency outcomes and that deterioration becomes more apparent when endoscopy is deferred beyond 24 hours [36]. Overall, the direction of effect supports ≤24-hour endoscopy as the pragmatic default, with very urgent endoscopy (≤6-12 hours) best reserved for selected high-risk presentations rather than applied routinely (Figure 2).

Outcomes

Efficiency outcomes were the most consistently improved when endoscopy was performed within 24 hours. Mortality benefits were most apparent when comparing endoscopy within 24 hours versus after 24 hours, supporting prioritization of early (≤24 hours) endoscopy in routine pathways, particularly in large administrative datasets such as Weissman et al. [18] and Garg et al. [26].

Impact on Mortality

Large-scale analyses reported higher mortality when endoscopy was delayed beyond 24 hours, with lower inpatient mortality when endoscopy occurred within 24 hours [18,26]. Similar “harm-with-delay” signals were also reported in cohort-level analyses [17,16]. In contrast, several hospital-based cohorts and audits did not demonstrate a clear mortality difference across earlier timing strata, despite reporting efficiency gains [30,24]. Comparable “no mortality difference” findings were also reported in the studies by Arslan et al. [25] and Iqbal et al. [31].

Elderly-Focused Cohorts

Findings in elderly-focused cohorts were mixed. In Cooper et al.'s study, early endoscopy was associated with favorable efficiency outcomes and lower surgery rates without a consistent mortality advantage [22]. In contrast, El-Dallal et al. reported shorter length of stay and lower charges with early endoscopy but higher adjusted mortality, suggesting residual confounding and selection effects in real-world timing decisions [23]. Overall, the most consistent survival signal relates to avoiding delays beyond 24 hours, while very early timing (≤6-12 hours) rarely adds mortality benefit beyond timely endoscopy within 24 hours.

Rebleeding and Therapeutic Interventions 

Evidence for rebleeding was heterogeneous, likely reflecting differences in baseline risk, timing thresholds, and case-mix. In Guo et al.'s study, very urgent endoscopy (<6 hours) was associated with a higher likelihood of repeat therapeutic endoscopy compared with 6-24 hours, a pattern plausibly driven by confounding by indication [16]. In high-risk cohorts, urgent timing (<12 hours) versus early timing (12-24 hours) did not consistently change rebleeding outcomes [29].

For intervention intensity and transfusion-related outcomes, some cohorts reported higher rates of endoscopic therapy with earlier endoscopy while maintaining similar recurrent bleeding and mortality profiles [19]. Other real-world cohorts did not demonstrate consistent differences in transfusion requirements or additional hemostasis across timing strata, indicating that earlier timing does not uniformly translate into improved hemostatic endpoints [24].

Pooled evidence also indicates that endoscopy within 24 hours does not consistently reduce mortality overall, and observational subgroup findings suggest very early endoscopy (within 12 hours) may be associated with higher mortality, underscoring the influence of baseline severity and residual confounding in timing comparisons [36].

Health-System Outcomes

Health-system efficiency outcomes were the most consistent domain across the evidence base. In large administrative datasets, endoscopy within 24 hours was associated with shorter length of stay and lower costs or reduced healthcare utilization [18,26].

Length-of-stay improvements were also observed in other observational designs and multicentre audit data, supporting early endoscopy as a practical throughput target in routine pathways [25,32].

However, efficiency gains were not universal. Some single-centre retrospective studies reported no difference in length of hospitalization by timing strata, despite differences in procedure burden (e.g., increased second-look endoscopy after very urgent timing) [28] and no consistent reduction in length of stay in other retrospective designs [31].

In older populations, earlier endoscopy was also linked to more efficient care delivery in population-based data [22].

Special Populations

Across elderly cohorts, early endoscopy generally aligned with efficiency gains, although residual confounding remains a concern. Cooper et al. [22] showed improved efficiency without a consistent mortality benefit, whereas El-Dallal et al. [23] reported shorter stay/charges but higher adjusted mortality, suggesting confounding by severity and selection.

Among anticoagulant-exposed NVUGIB patients, early endoscopy was feasible and was associated with shorter stays and higher therapeutic intervention rates without clear mortality differences [19].

Integration of Evidence

Higher-level evidence indicates that UGIB outcomes reflect both endoscopy timing and key co-interventions within the care pathway. Villanueva et al. showed that a restrictive transfusion strategy improved survival and reduced further bleeding compared with a liberal strategy, highlighting the importance of concurrent pathway choices when interpreting timing effects [33].

Across pooled timing analyses, early endoscopy within 24 hours was most consistently associated with efficiency improvements, while a consistent mortality reduction was not demonstrated overall. Observational subgroup findings also suggest very early endoscopy (within 12 hours) may be associated with higher mortality, again emphasizing confounding by severity and the need for risk-stratified decision-making [36].

Table 2 gives a summary of key outcome patterns across mortality, rebleeding/therapeutic interventions, health system efficiency, and special populations.

**Table 2:** Summary of outcome patterns across objectives in the included studies of NVUGIB, stratified by outcome domain and timing threshold For each outcome domain, the supporting studies are listed in the Studies column (citation numbers correspond to the reference list) and are not repeated in the Main findings column. Timing is summarized primarily as early endoscopy (≤24 hours) versus delayed endoscopy (>24 hours), with urgent thresholds (≤12 hours and ≤6 hours) noted where reported. NVUGIB, non-variceal upper gastrointestinal bleeding; LOS, length of stay; RCT, randomized controlled trial; OR, odds ratio; CI, confidence interval; EGD, esophagogastroduodenoscopy.

Category	Studies	Main findings
Mortality outcomes	Guo et al. [16]; Jeong et al. [17]; Weissman et al. [18]; Garg et al. [26]; Tripathi et al. [21]; Cooper et al. [22]; El-Dallal et al. [23]; Chang et al. [24]; Arslan et al. [25]; Cagir et al. [27]; Saleem et al. [28]; Jairath et al. [30]; Iqbal et al. [31]; Barbu et al. [34].	Large database studies generally reported lower inpatient mortality with endoscopy within ≤24 hours and worse outcomes with delays beyond 24 hours. Cohort and audit studies were mixed, with several reporting no clear mortality difference across early timing strata. Elderly-focused evidence was conflicting: some analyses showed efficiency gains without clear mortality benefit, while one geriatric analysis reported higher adjusted mortality despite shorter stay/charges, suggesting residual confounding. Overall, the most consistent mortality signal relates to avoiding delays beyond 24 hours, whereas very urgent windows (≤6–12 hours) rarely add consistent survival advantage.
Rebleeding and therapeutic interventions	Guo et al. [16]; Capela et al. [19]; Chang et al. [24]; Güven et al. [29]; Siau et al. [32]; Bai et al. [36].	Evidence for rebleeding is heterogeneous across timing thresholds and risk strata. In some datasets, very urgent timing (e.g., <6 hours) was associated with more repeat therapeutic endoscopy (repeat-procedure pattern), likely reflecting confounding by severity and indication rather than causal harm. In high-risk cohorts, comparisons of <12 hours vs 12–24 hours did not consistently change rebleeding. Earlier endoscopy was associated with higher rates of endoscopic therapy in some populations (notably anticoagulant-exposed cohorts). Meta-analytic evidence supports consistent efficiency gains with ≤24-hour endoscopy, while mortality and bleeding endpoints vary and may be influenced by baseline severity and timing definitions.
Health-system outcomes (LOS, costs, discharge/throughput)	Guo et al. [16]; Weissman et al. [18]; Capela et al. [19]; Garg et al. [26]; Tripathi et al. [21]; Cooper et al. [22]; El-Dallal et al. [23]; Chang et al. [24]; Arslan et al. [25]; Cagir et al. [27]; Saleem et al. [28]; Siau et al. [32]; Iqbal et al. [31].	Efficiency outcomes were the most consistent domain overall, particularly in large datasets where ≤24-hour endoscopy was associated with shorter LOS and/or lower costs/charges. Efficiency gains were also reported in anticoagulant-exposed patients and several elderly-focused analyses. However, efficiency benefits were not universal: some hospital-based cohorts reported no LOS difference, and at least one cohort reported higher LOS/costs with early timing, highlighting variation by setting, case-mix, and local pathways. Overall, timely ≤24-hour endoscopy remains a pragmatic throughput target, with expected efficiency benefits in many (but not all) contexts.
Special populations	Capela et al. [19]; Cooper et al. [22]; El-Dallal et al. [23]; Cagir et al. [27].	Elderly: early endoscopy generally aligned with efficiency gains, but mortality signals were inconsistent, and one adjusted analysis suggested higher mortality despite efficiency improvements, consistent with residual confounding. Anticoagulant-exposed NVUGIB: early endoscopy was feasible and associated with shorter LOS and higher therapeutic intervention rates, without consistent mortality differences.
Integrated evidence	Villanueva et al. [33]; Bai et al. [36].	Higher-level evidence indicates outcomes depend on timing and co-interventions within the UGIB pathway. RCT evidence supports improved outcomes with a restrictive transfusion strategy, emphasizing the importance of supportive-care decisions when interpreting timing effects. Meta-analytic synthesis supports consistent efficiency improvements with ≤24-hour endoscopy, while mortality benefit is not consistently demonstrated across all comparisons and may be most apparent when contrasted against >24-hour delays, with very early windows potentially reflecting confounding by baseline severity in observational cohorts.

Risk of Bias Assessment

RCTs were assessed using the Cochrane Risk of Bias 2 (RoB 2) tool. Cohort studies, propensity score-matched cohorts, and prospective observational studies were appraised using the Newcastle-Ottawa Scale (NOS). Nationwide administrative database studies were evaluated using adapted NOS criteria emphasizing selection, comparability (confounding control), and outcome ascertainment. Audit-based studies were assessed using NOS-aligned criteria emphasizing representativeness and reporting completeness.

**Figure 3:**
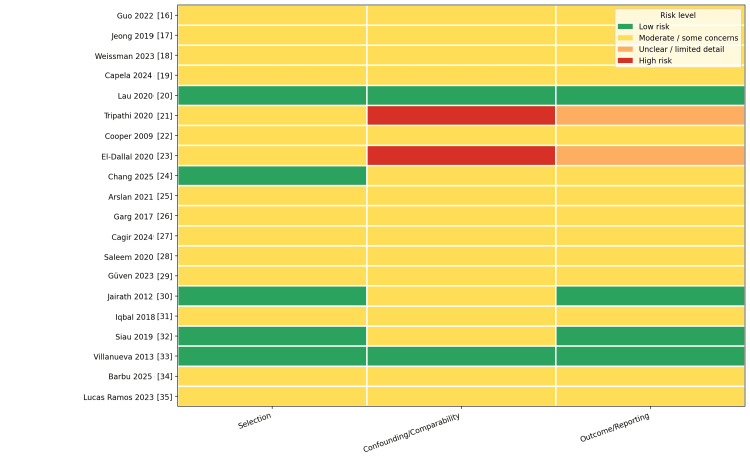
Risk of bias and quality assessment of included studies Risk levels are shown by color: green = low risk; yellow = moderate risk/some concerns; orange = unclear risk/limited methodological detail; red = high risk. Domains assessed were selection, confounding/comparability, and outcome/reporting. DB, administrative database; PSM, propensity score matching; RCT, randomized controlled trial.

Risk of bias was judged across selection, confounding/comparability, and outcome/reporting, then summarized as low risk, moderate risk (some concerns), unclear risk (limited methodological detail), or high risk of bias. Most observational studies were rated as moderate risk, most commonly due to residual confounding/comparability limitations; the RCTs were rated as low risk. Audit studies were generally low risk for selection and outcome/reporting but retained some concerns for comparability. Abstract-only reports were rated as unclear or of greater concern because methodological detail and confounding control could not be fully verified. Figure 3 summarizes the risk of bias and quality assessment across the included studies.

Interpretation

Most nationwide database studies were rated as moderate risk overall, primarily due to residual confounding inherent to non-randomized administrative data [18,26]. Cohort studies were similarly rated as moderate risk, driven mainly by confounding control and, secondarily, outcome ascertainment [16,17]. Abstract-only reports carried the highest concerns/unclear risk due to limited methodological detail [21,23]. Audit-based studies were typically limited by incomplete confounding adjustment but were adequately reported for service-level outcomes [32].

Discussion

This systematic review synthesized evidence from 20 studies, including more than 4.4 million NVUGIB patients. Overall, early endoscopy (≤24 hours) was most consistently associated with improved efficiency outcomes (shorter length of stay, lower costs, improved throughput), particularly in large administrative datasets [18,26]. Mortality benefits were clearest when comparing ≤24 hours versus >24 hours, whereas smaller hospital-based cohorts more often reported no clear survival difference [16,25]. Very urgent endoscopy (≤6-12 hours) rarely provided incremental survival benefit beyond a timely ≤24-hour endoscopy, except potentially in selected high-risk presentations [20,29].

Comparison With Existing Literature

These findings align with pooled evidence indicating that ≤24-hour endoscopy improves efficiency outcomes and that deterioration becomes more evident when endoscopy is delayed beyond 24 hours [36,37]. Large-scale analyses consistently reported lower inpatient mortality and reduced resource use with early endoscopy [18,26]. Territory-wide and cohort studies suggest that the principal harm signal is driven by delays beyond 24 hours rather than small differences within the early window [16,17]. Evidence also cautions against indiscriminate “ultra-urgent” endoscopy in lower-risk patients, where early clot manipulation or incomplete stabilization may contribute to higher rebleeding or repeat procedures [16,38]. This is supported by Kim et al.'s study, where urgent endoscopy within six hours did not reduce 30-day mortality, recurrent bleeding, transfusion requirements, or length of stay compared with endoscopy performed after six hours in an emergency-department UGIB cohort [39]. Similar timing-outcome patterns have been observed in other large NVUGIB cohorts [40]. Overall, the data support a risk-based strategy: prioritize ≤24-hour endoscopy broadly and reserve ≤6-12-hour timing for patients with clear high-risk features.

Interpretation of Results

Clinically, most NVUGIB patients can be managed safely with prompt resuscitation and endoscopy within 24 hours, supported by validated risk stratification to determine urgency. Routine procedures within 6-12 hours are unlikely to be necessary unless patients have hemodynamic instability, ongoing bleeding, very high risk scores, or other high-risk markers [29,41]. In elderly and anticoagulant-exposed populations, early endoscopy appears feasible and generally efficient, although conflicting mortality signals in some datasets highlight ongoing residual confounding concerns [22,23]. Anticoagulant-focused evidence suggests early endoscopy can be delivered without a clear mortality penalty, with shorter stays and higher therapeutic intervention rates in some cohorts [18,19].

Implications for Practice

These findings support pathways that prioritize endoscopy within 24 hours for NVUGIB, reserving urgent timing for selected high-risk cases rather than applying it universally. Services should emphasize rapid stabilization, clear triage, and dependable access to endoscopy within the first day of presentation. Because disparities in access to endoscopy can influence outcomes at a systems level, pathway design should also consider equity and referral barriers.

Strengths and Limitations

Strengths include PRISMA-aligned selection, inclusion of diverse study designs, and incorporation of large-scale datasets supporting external validity [18,26]. Limitations are primarily heterogeneity and residual confounding: studies differed in case-mix, timing definitions (≤6, ≤12, ≤24 hours), and adjustment approaches, complicating direct comparisons [16,30]. Abstract-only reports provided limited methodological detail and therefore carried higher risk-of-bias concerns [21,23]. No quantitative pooling was performed; therefore, conclusions rely on the direction and consistency of findings rather than meta-analytic summary estimates.

Future Directions

Future work should prioritize pragmatic multicenter trials or strong quasi-experimental designs comparing urgent versus early strategies in clearly defined high-risk NVUGIB populations, using standardized timing definitions and risk-score stratification [20,29]. Studies should more consistently report protocol co-interventions (e.g., transfusion strategy and hemostasis approach), as outcomes likely reflect combined pathway effects rather than timing alone [33,36]. Additional focused evidence is needed in elderly and anticoagulant-exposed groups, where the balance between safety, efficiency, and confounding remains especially challenging [19,23].

Summary

Overall, the evidence supports endoscopy within 24 hours as the most reliable strategy to improve efficiency outcomes in NVUGIB and to avoid harm associated with delays beyond 24 hours [17,18]. Very urgent endoscopy (≤6-12 hours) should be targeted to patients at the highest clinical risk, since routine ultra-urgent timing offers limited additional survival benefit in unselected populations [16,20].

## Conclusions

This systematic review indicates that, for most patients withsuspected NVUGIB and no contraindications, endoscopy within 24 hours improves clinical efficiency by shortening hospital stay, reducing costs, and supporting better discharge outcomes, while offering the clearest survival advantage when compared with delays beyond 24 hours. Urgent endoscopy within 6-12 hours should be reserved for patients who are hemodynamically unstable, very high risk, or receiving anticoagulant therapy, with timing individualized when endoscopy is temporarily unsafe or not feasible.

Achieving these targets depends on timely diagnosis and referral across emergency and inpatient services together with adequate endoscopy capacity. Future research should prioritize multicenter trials using standardized timing definitions and formal cost-effectiveness evaluations to clarify the resource implications of expanding timely endoscopy.
